# Prognostic value of plasma diquat concentration in patients with acute oral diquat poisoning: a retrospective study

**DOI:** 10.3389/fpubh.2024.1333450

**Published:** 2024-06-04

**Authors:** Na Meng, Yiqing Sun, Yanling Dong, Baopu Lv, Dongqi Yao, Hengbo Gao, Yu Ma, Yingli Jin, Tieying Zhu, Yingping Tian

**Affiliations:** Department of Emergency, The Second Hospital of Hebei Medical University, Shijiazhuang, China

**Keywords:** diquat, poisoning, plasma DQ concentration on admission, prognosis, time-dependent Cox regression analyses

## Abstract

**Objectives:**

Diquat poisoning is an important public health and social security agency. This study aimed to develop a prognostic model and evaluate the prognostic value of plasma diquat concentration in patients with acute oral diquat poisoning, focusing on how its impact changes over time after poisoning.

**Methods:**

This was a retrospective cohort study using electronic healthcare reports from the Second Hospital of Hebei Medical University. The study sample included 80 patients with acute oral Diquat poisoning who were admitted to the hospital between January 2019 and May 2022. Time-to-event analyses were performed to assess the risk of all-cause mortality (30 days and 90 days), controlling for demographics, comorbidities, vital signs, and other laboratory measurements. The prognostic value of plasma DQ concentration on admission was assessed by computing the area under a time-dependent receiver operating characteristic curve (ROC).

**Results:**

Among the 80 patients, 29 (36.25%) patients died, and 51 (63.75%) patients survived in the hospital. Non-survivors had a median survival time (IQR) of 1.3(1.0) days and the longest survival time of 4.5 days after DQ poisoning. Compared with non-survivors, survivors had significantly lower amounts of ingestion, plasma DQ concentration on admission, lungs injury within 24 h after admission, liver injury within 24 h after admission, kidney injury within 24 h after admission, and CNS injury within 36 h after admission, higher APACHE II score and PSS within 24 h after admission (all *p* < 0.05). Plasma Diquat concentration at admission (HR = Exp (0.032–0.059 × ln (t))) and PSS within 24 h after admission (HR: 4.470, 95%CI: 1.604 ~ 12.452, *p* = 0.004) were independent prognostic factors in the time-dependent Cox regression model.

**Conclusion:**

Plasma DQ concentration at admission and PSS within 24 h after admission are independent prognostic factors for the in-hospital case fatality rate in patients with acute oral DQ poisoning. The prognostic value of plasma DQ concentration decreased with time.

## 1 Introduction

Diquat (1,1′-ethylene-2,2′-bipyridinium ion; DQ) is a type of bipyridinium herbicide that acts similarly to paraquat (PQ) but has distinct poisoning mechanisms and clinical effects. Diquat is a highly toxic herbicide that is commonly used to control weeds in agricultural and non-agricultural settings. Its usage has increased, particularly in developing countries where paraquat has been banned, leading to a steady increase in DQ poisoning cases (1, 2). DQ poisoning is often caused by unintentional or deliberate ingestion of concentrated liquid products containing DQ, leading to multiple organ dysfunction and even death. Based on previous studies, in some cases, the fatality rate for DQ poisoning can be as high as 52.5% (3). After PQ poisoning, timely treating DQ poisoning has become another problematic task for clinicians. Therefore, effective evaluation of clinical outcomes and risk assessment for critically ill DQ poisoning patients is crucial for the optimal allocation of medical resources and for improving public health and social security. This requires timely diagnosis, monitoring, and treatment to prevent serious complications and improve survival rates. Clinicians should have access to updated guidelines, treatment protocols, and appropriate resources and equipment to manage DQ poisoning cases. Education and awareness programs for the public can also help to prevent DQ poisoning and promote early intervention.

The severity of DQ poisoning is divided into mild poisoning, moderate to severe poisoning, and fulminant poisoning. The survival probability of the patients is roughly evaluated based on the dose ingested (DQ2+) and different clinical manifestations (3, 4). Ingestion of diquat in large amounts can lead to severe toxicity and life-threatening complications. Although the exact lethal dose of DQ in humans is not well-defined, it is estimated to be around 20–30 mg/kg of body weight. Ingestion of a significant amount of DQ, such as 20–50 mL of a concentrated formulation, can cause severe toxic effects on various organs, including the liver, kidney, and lungs. For example, patients with higher plasma DQ concentrations on admission have been shown to have a significantly higher mortality rate and longer hospital stay. Ingestion of a large amount of DQ can lead to multi-organ failure and cardiogenic shock within 1–4 days, which can result in death. Previous studies have found that the plasma DQ concentration can be a useful biomarker for predicting the severity of DQ poisoning and monitoring the efficacy of treatment. However, the total dose absorbed (DQ2+) varies widely due to subjective expression, variable gastrointestinal absorption function, and different gastric lavage and catharsis intervention timings. Poison detection is a crucial component of acute poisoning clinical diagnosis. Plasma poison concentration has great value in evaluating the prognosis and guiding treatment. By analyzing the plasma DQ concentration in patients with DQ poisoning, healthcare professionals can determine the appropriate dosage of antioxidant therapy and adjust treatment strategies accordingly. Additionally, measuring plasma diquat concentration can provide important information for medical professionals to assess the prognosis of patients with acute DQ poisoning. Previous studies indicated, even smaller quantities of diquat ingestion, such as 10–20 mL, can cause irreversible lung fibrosis and renal failure that would result in death within several weeks. Therefore, the ingestion volume and plasma concentration of diquat are often used as important indicators of patients’ prognosis. These studies also highlight the importance of monitoring plasma diquat concentration in patients with acute DQ poisoning to optimize treatment and improve patient outcomes. For example, Hart et al. created concentration-time curves to represent estimates of the survival probability of acute PQ poisoning (5). Hampson et al. found that when plasma PQ concentrations were higher than 3 mg/L, the patient had a poor prognosis despite hemoperfusion (6).

However, the prognostic value of plasma DQ concentration in patients with acute DQ poisoning is still lacking solid evidence due to the various limitations, such as sample size, methodology, etc. For example, statistical techniques like regression analysis, survival analysis, or logistic regression may be used to determine if there’s a significant relationship between plasma diquat levels and patient prognosis, such as mortality rates, severity of poisoning, or recovery times. However, traditional models might implicitly assume that this condition is met, which can lead to biased or incorrect results if the assumption is violated. Therefore, this study aimed to identify prognostic factors of patients with acute oral DQ poisoning and evaluate the prognostic value of plasma DQ concentration at admission using statistical tests and graphical diagnostics based on scaled Schoenfeld residuals, which could contribute to clinical evaluation and treatment. In addition, this approach could provide a more rigorous and objective assessment of whether the proportional hazards assumption holds true for the data. By using scaled Schoenfeld residuals, researchers can visually inspect and statistically test for any time-dependent covariates, ensuring the model’s assumptions are valid and its conclusions are more reliable (7).

## 2 Materials and methods

### 2.1 Study sample

We performed a retrospective cohort study with a population from the Second Hospital of Hebei Medical University electronic healthcare records (HER) dataset to identify all patients who were treated as DQ poison from January 1, 2019, to May 31, 2022. The EHR dataset includes information on socioeconomic characteristics, diagnoses, medications, procedures, laboratory and imaging reports, vital signs, the length of hospitalizations, and death date. Plasma samples were collected at admission. Plasma DQ concentration was measured by high-performance liquid chromatographic tandem mass spectrometry (HPLC-MS/MS, Shimadzu, Japan; AB Sciex, United States). Separation of analytes was achieved on a Pc Hilic S5 column (5 μm, 2.0 mm × 150 mm) (Osaka Soda, Beijing) at 35°C. The mobile phase consisted of a mixture of solvent A (20 mM ammonium formate in water containing 0.1% formic acid) and solvent B (acetonitrile) delivered with a gradient at a flow rate of 0.3 mL/min. The standard curve was linear over a plasma concentration range of 10–1,000 ng/mL. The study followed the Strengthening the Reporting of Observational Studies in Epidemiology (STROBE) reporting guideline (8). The exemption from obtaining written informed consent was granted due to the retrospective observational nature of our study. The data analysis was performed from June 1, 2022, to July 31, 2022. The Hebei Medical University Institutional Review Board reviewed and approved the study (IRB: 2020-C043).

This study analyzed admitted patients at the Second Hospital of Hebei Medical University who were diagnosed with DQ poisoning. To be included, patients had to have a documented history of oral DQ ingestion, be at least 14 years old, have DQ detected in plasma and/or urine samples taken upon admission, and have a time interval from DQ ingestion to ED admission of no more than 36 h. Patients were excluded if they had mixed toxicant poisoning, non-oral exposure routes, blood purification in local hospitals, or a history of severe lung disease or severely impaired liver or kidney function. The final study sample consisted of 392 adults.

All patients received immediate gastric lavage, activated charcoal absorption, and diarrhea induction with purgative and/or high enemas to prevent DQ absorption. Forced diuresis, hemoperfusion (HP), and/or hemofiltration were applied to promote DQ excretion. Blood purification was administered to patients within 1–2 h after admission. The old therapeutic regimen referred to patients who received 2 h of HP at 6 to 8 h interval time. The HP frequency was adjusted according to the plasma DQ concentration. The new therapeutic regimen adopts the model of “HP + Continuous Veno-Venous Hemofiltration (CVVH) +” The interval between two HP was 9 to 10 h, during which a CVVH was applied. The HP and CVVH frequencies were adjusted according to the plasma DQ concentration. Antioxidants (vitamin C and melatonin) and low-dose glucocorticoids were used to scavenge free radicals and inflammatory mediators. Other clinical treatments included maintaining fluid and electrolyte balance, organ function support, etc. (3, 4).

### 2.2 Measurements

The primary outcome of interest was all-cause in-hospital mortality. To identify the overall mortality rate among patients with acute DQ poisoning, and considering a null hypothesis proportion of 0.3, a true proportion of 0.5, a type I error rate of 5%, and a desired power of 80%, a sample size of 49 patients was required for this study.

The secondary outcomes of interest were the occurrence of complications after acute DQ poisoning, including DQ induced lung injury, liver injury, kidney injury, and central nervous system (CNS) injury. Specifically, the lung injury was identified based on the following criteria for (9), (1) history of DQ ingestion that is known to induce lung injury, (2) clinical manifestations have been reported to be induced by DQ, (3) Other causes of clinical manifestations could be ruled out, (4) partial pressure oxygen (PaO2) < 80 mmHg on room air. The liver injury was proposed if one of the following thresholds was met (10) (1) alanine aminotransferase (ALT) ≥ 5 × upper limits of normal (ULN), (2) alkaline phosphatase (ALP) ≥ 2 × ULN (especially with the elevation of gamma-glutamyl transferase (GGT) or after ruling out primary bone pathology in cases of isolated elevation of ALP), (3) ALT ≥3 × ULN plus total bilirubin (TB) > 2 × ULN. The kidney injury was proposed if one of the following thresholds was met (11, 12) (1) serum creatinine increased 1.5 times baseline, (2) urine output <0.5 mL/kg/h during a 6-h block. The CNS injury was proposed if one of the following thresholds was met (3, 13) (1) Clinical manifestations with headache, dizziness, disturbance of consciousness (drowsiness, confusion of consciousness, delirium, lethargy, coma), focal or generalized epileptiform seizures, etc., (2) Brain imaging can manifest as cerebral edema, brain stem infarction, and bleeding.

### 2.3 Covariates

The covariates of patients with DQ poisoning included baseline demographics (age and sex), the length of gastric lavage, ED stay, and blood purification. We also measured the scores of the Acute Physiology and Chronic Health Evaluation (APACHE II) and poisoning severity score (PSS) within 24 h of admission (14, 15), treatment regimens, frequency of hemoperfusion (HP), frequency of continuous veno-venous hemofiltration (CVVH) and hospital days.

### 2.4 Statistical analyses

Baseline patient characteristics were compared using the Chi-square test for categorical variables and student t-test for continuous variables across survival and non-survival patients with DQ poisoning.

Then we conducted two univariate Cox proportional hazards regression was used to assess associations, measured as hazard ratios (HRs), between covariates and time. Variables that showed a *p* value less than 0.1 were included in multivariate Cox proportional hazards regression analyzes. The proportional hazards assumption was checked using the Schoenfeld residuals method (16). The linearity of continuous covariates was checked using the Martingale residuals method. In situations when the proportional hazards assumption of the Cox regression model does not hold, introducing a time-dependent variable (T_COV_) in Cox proportional hazards regression analyzes provided a flexible method to evaluate non-proportionality. Specifically, the Schoenfeld residuals were used to verify the assumption of proportional hazards. Considering that time variation generally corresponds to a skewed distribution, the natural logarithm of the time variable was used to build time-dependent covariates in the time-dependent Cox regression model to reduce the influence of extreme values [26]. The hazard ratio (HR) and the 95% confidence interval (95% CI) were calculated.

The prognostic value of plasma DQ concentration on admission was assessed by computing the area under a time-dependent receiver operating characteristic curve (ROC). The optimal cutoff value represented the highest Youden index (sensitivity + specificity-1). Survival curves were generated using the Kaplan–Meier method and compared using the log-rank test. A *p* value less than 0.05 was considered statistically significant. SPSS 22.0 software (SPSS, Inc., Chicago, IL, United States) and R software (version 3.3.0, R Foundation for Statistical Computing, Vienna, Austria) were used for statistical analysis.

## 3 Results

**Figure 1:**
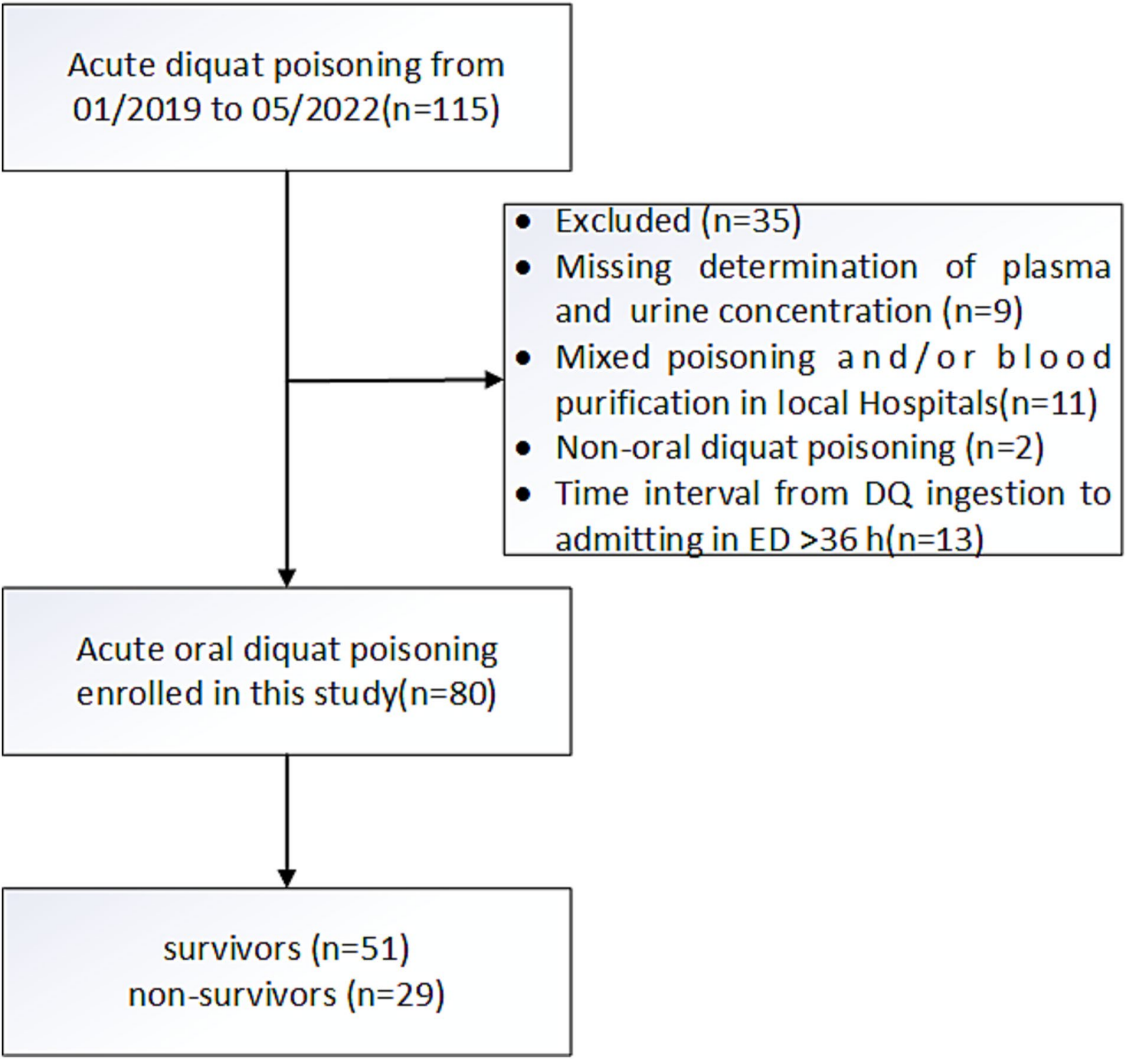
Flowchart of the study cohorts.

A total of 80 patients who met the criteria were included in the study (Figure 1). Seventy-eight patients took concentrated liquid products containing 200 g/L of DQ and 2 patients took concentrated liquid products containing 100 g/L of DQ. For recording, all patients who reproduced the amount of ingestion were converted into concentrated liquid products containing 200 g/L of DQ. The baseline demographics and clinical characteristics of patients with acute oral DQ poisoning are shown in Table 1. Among the 80 patients, 29 (36.25%) patients died, and 51 (63.75%) patients survived in the hospital. Non-survivors had a median survival time (IQR) of 1.3(1.0) days and the longest survival time of 4.5 days after DQ poisoning. Between survivors and non-survivors, there were no significant differences in gender, age, time interval from DQ ingestion to gastric lavage, time interval from DQ ingestion to ED, time interval from DQ ingestion to blood purification, treatment regimens (all *p* > 0.05). But survivors had significantly lower amounts of ingestion, plasma DQ concentration on admission, lungs injury within 24 h after admission, liver injury within 24 h after admission, kidney injury within 24 h after admission, and CNS injury within 36 h after admission (all *p* < 0.05). Furthermore, traditional score comparisons between survivors and non-survivors also showed that non-survivors had a significantly higher APACHE II score and PSS within 24 h after admission (all *p* < 0.05).

**Table 1:** Baseline demographics and clinical characteristics of the diquat poisoning.

Variable	Total (*N* = 80)	Survivors (*N* = 51)	Non-survivors (*N* = 29)	*p*-value
Age(years)	26.67 ± 9.73	26.08 ± 15.0	27.72 ± 12.5	0.47
Male, n (%)	37 (46.3%)	21 (41.2%)	16 (55.2%)	0.23
Ingestion amount (mL)	50 (80)	20 (40)	100 (137.5)	<0.01*
Time to gastric lavage(h)	2 (3)	2 (4)	1 (2)	0.092
Time to our ED(h)	6 (5)	6 (5)	6 (5)	0.912
Time to blood purification (h)	8.5 (5)	9 (5)	8.5 (5)	0.912
PDQ (ug/mL)	1.06 (9.57)	0.35 (0.90)	26.9 (46.65)	<0.01*
**Organ injury**				
Lungs injury_24-h_, n (%)	17 (21.3%)	3 (5.9%)	14 (48.3%)	<0.01*
Liver injury_24-h_, n (%)	6 (7.5%)	1 (2.0%)	5 (17.2%)	0.04*
Kidney injury_24-h_, n (%)	31 (38.8%)	5 (9.8%)	26 (89.7%)	<0.01*
CNS injury_36-h_, n (%)	28 (35.0%)	4 (7.8%)	24 (82.8%)	<0.01*
APACHE-II _24-h_	6 (8)	4 (4)	13 (12)	<0.01*
PSS _24-h_	1 (2)	1 (1)	4 (1)	<0.01*
**Treatment regimens**				0.17
New regimens	57 (71.3%)	39 (76.5%)	18 (62.1%)	
Old regimens	23 (28.8%)	12 (23.5%)	11 (37.9%)	
Survival Time (d)	–	–	1.30 (1.00)	–

ED, emergency department; PDQ, Plasma DQ concentration on admission; CNS, central nervous system; APACHE-II, Acute Physiology and Chronic Health Evaluation; PSS, Poisoning severity score; HP, Hemoperfusion; CVVH, Continuous veno-venous hemofiltration; Old regimen, received 2-h of HP therapy, at a 6 to 8-h interval time. New regimen: the model of “HP + CVVH + HP.” The interval time between two HP was 9 to 10 h, during which a CVVH was applied. The frequency of HP and CVVH was adjusted according to the plasma DQ concentration.

*Statistically significant.

### 3.1 Proportional hazards assumption verification

**Figure 2:**
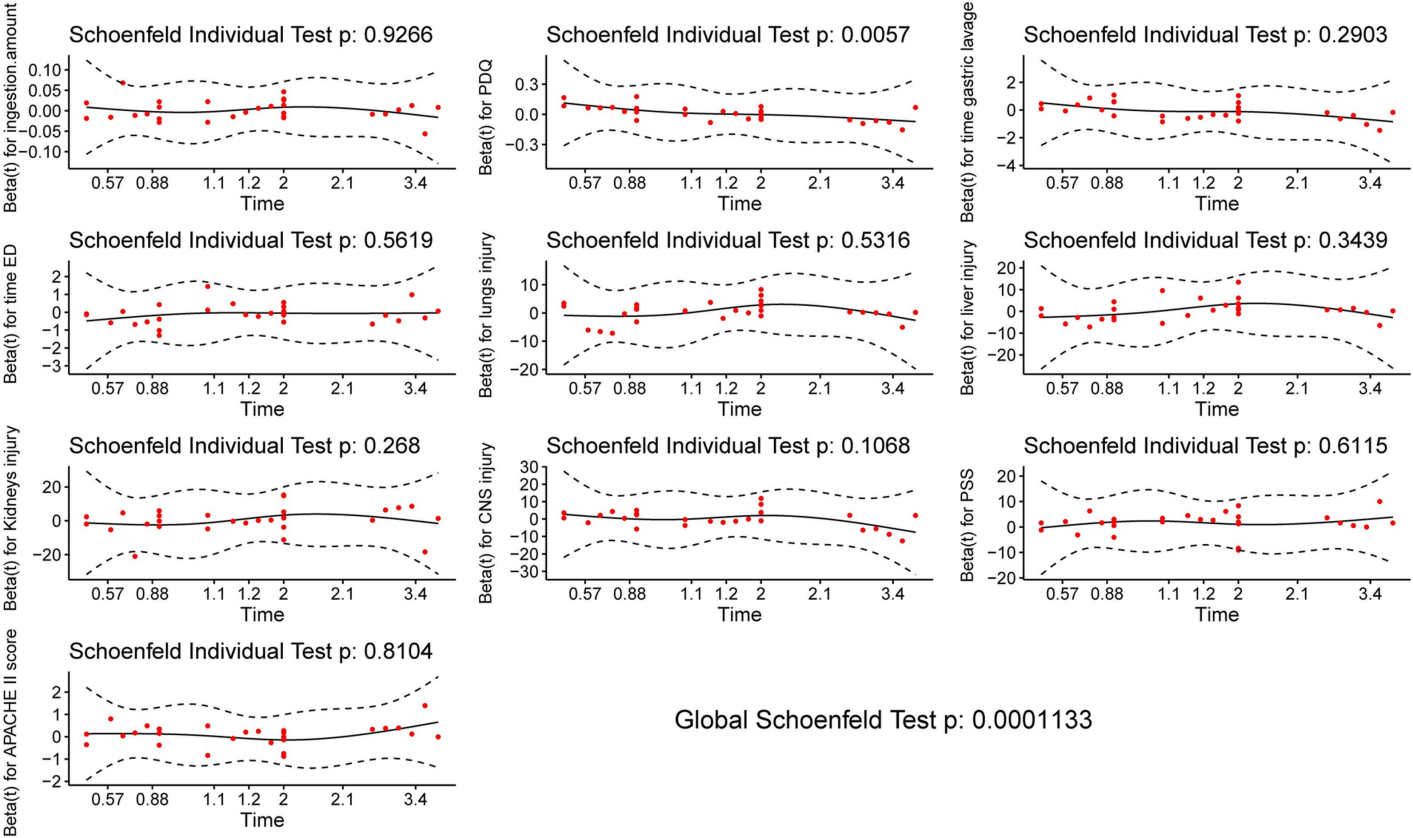
Proportional hazards assumption based on scaled schoenfeld residuals.

The proportional hazards assumption is verified using statistical tests and graphical diagnostics based on scaled Schoenfeld residuals. As shown in Figure 2, the Schoenfeld individual test is not statistically significant (*p* > 0.05) for the amount of ingestion, the time interval from DQ ingestion to gastric lavage, the time interval from DQ ingestion to the ED, lung injury within 24 h after admission, liver injury within 24 h after admission, kidney injury within 24 h after admission and CNS injury within 36 h after admission, but statistically significant (*p* < 0.05) for plasma DQ concentration at admission. The Schoenfeld residual of plasma DQ concentration at admission is evidence of a violation of the proportional hazard assumption. Furthermore, the global test shows a statistically significant (*χ*^2^ = 35.247, *p* = <0.001) correlation between the Schoenfeld residuals and the time variation, indicating that the Cox proportional hazards model has been shown to be inappropriate in multivariate analysis.

### 3.2 Univariate Cox proportional hazards regression analyses

According to the verification of the proportional hazards assumption, plasma DQ concentration at admission is a time-dependent covariate. Therefore, the time-dependent Cox regression model is appropriate for dissecting the influences of these time-dependent covariates. The results of the univariate Cox regression analysis are presented in Table 2. Univariate Cox regression analysis revealed that the amount of ingestion, plasma DQ concentration on admission, lung injury within 24 h after admission, liver injury within 24 h after admission, kidney injury within 24 h after admission, CNS injury within 36 h after admission, APACHE II score and PSS within 24 h of admission had statistical differences (all *p* < 0.01).

**Table 2:** Univariate Cox regression analysis.

Variable	Cofe	SE (cofe)	Z	HR (95%CI)
Age	0.012	0.016	0.740	1.012 (0.980,1.045)
Gender	0.395	0.374	1.057	1.488 (0.714,3.088)
Ingestion amount (mL)	0.011	0.002	6.519	1.011 (1.008,1.014)*
Time to gastric lavage	−0.078	0.062	−1.262	0.925 (0.818,1.044)
Time to our ED	−0.023	0.036	−0.632	0.978 (0.911,1.049)
PDQ (ug/mL)	0.068	0.010	7.131	1.071 (1.051,1.091)*
T_COV_ PDQ	−0.017	0.983	−1.119	0.983 (0.953,1.013)
Lungs injury_24-h_	1.883	0.383	4.915	6.577 (3.103,13.94)*
Liver injury_24-h_	1.357	0.500	2.713	3.883 (1.457,10.35)*
Kidney injury_24-h_	3.432	0.622	5.517	30.93 (9.139,104.6)*
CNS injury_36-h_	2.984	0.503	5.937	19.76 (7.379,52.91)*
APACHE-II_24-h_	0.117	0.015	7.798	1.124 (1.092,1.158)*
PSS _24-h_	1.837	0.272	6.746	6.277 (3.681,10.70)*
treatment regimens	−0.606	0.384	−1.579	0.546 (0.257,1.158)

ED, emergency department; PDQ, Plasma DQ concentration on admission; CNS, central nervous system; APACHE-II, Acute Physiology and Chronic Health Evaluation; PSS, Poisoning severity score.

*Statistically significant.

### 3.3 Multivariate Cox proportional hazards regression analyses

According to the univariate Cox regression analysis, ingestion amount, plasma DQ concentration at admission, lungs injury within 24 h after admission, liver injury within 24 h after admission, kidney injury within 24 h after admission, CNS injury within 36 h after admission, APACHE II score and PSS within 24 h after admission are included in the multivariate Cox regression analysis. The time interval from DQ ingestion to gastric lavage and the time interval from DQ ingestion to the emergency department are critical in assessing in-hospital deaths from acute DQ poisoning and are therefore also included in the multivariate Cox regression analysis. There was no multicollinearity among the above indicators. This study satisfies the hypothesis of a linear relationship between continuous variables and the outcome.

Time-dependent multivariate Cox proportional hazards regression analyzes revealed that plasma DQ concentration at admission, T_COV_PDQ, and PSS within 24 h after admission are statistically significant (*p* < 0.05), as shown in Table 3. Plasma DQ concentration at admission and T_COV_PDQ are statistically significant, implying that the effect of plasma DQ concentration at admission varies with time. The time-varying effect of plasma DQ concentration at admission can be written as β (t) =0.032–0.059 × ln (t) and HR (t) = Exp (0.032–0.059 × ln (t)). With 1.5 days (36 h) after poisoning, the HR of plasma DQ concentration on admission gradually decreased with time.

**Table 3:** Multivariate Cox proportional hazards regression analyses.

Variable	Cofe	se(cofe)	Z	*P*-value	HR(95%CI)
PDQ (ug/mL)	0.032	0.016	2.049	0.040	1.033 (1.001,1.066)*
T_COV_ PDQ	−0.059	0.024	−2.520	0.0122	0.942 (0.900,0.987)*
PSS _24-h_	1.497	0.523	2.864	0.004	4.470 (1.604,12.452)*

PDQ, Plasma DQ concentration on admission; PSS, Poisoning severity score.

*Statistically significant.

### 3.4 Comparison of the impact of plasma DQ concentration on admission and ingestion amount on prognosis using time-dependent ROC analysis

**Figure 3:**
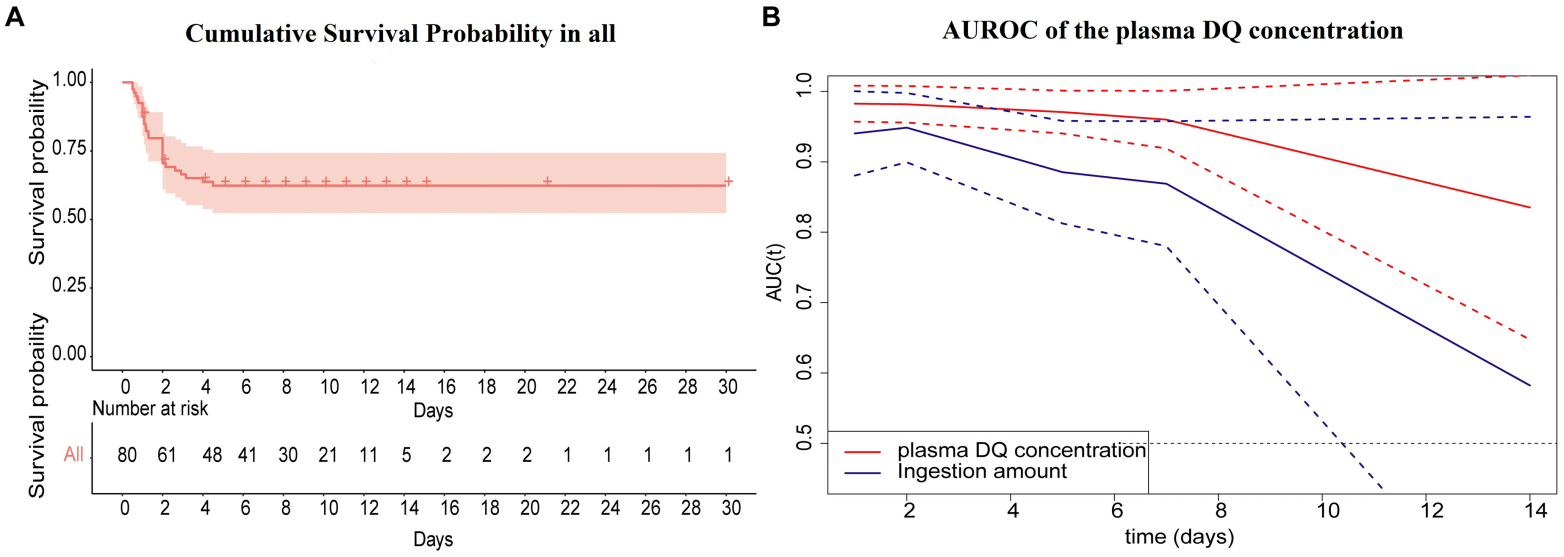
**(A, B)** The cumulative survival probability in all cases after diquat poisoning (primary analyses).

The cumulative survival probability in all cases was shown in Figure 3A. The cumulative survival probability of 1, 2, 4 and 4.5 days in all cases was 87.5, 70.5, 63.7 and 62.3%, respectively. The median survival time was 19.33 days (95%CI 16.256–22.410). No events occurred beyond 5 days in this study. We conducted a time-dependent ROC analysis to assess the impact of plasma DQ concentration on admission and the amount of ingestion on the prediction of survival or death from DQ poisoning in patients. In this study, according to the time characteristics of the death event, we computed the time dependent AUCs (95%CI) to evaluate their predictive precision at 1, 2, 5, 7, and 14 days, respectively (Table 4). Continuously changing AUCs and confidence intervals as days after poisoning were drawn using the plotAUCcurve function, as shown in Figure 3B. These results revealed that the AUROC of the plasma DQ concentration on admission and ingestion amount decreased with days after poisoning. In contrast, the AUROC of the plasma DQ concentration at admission was higher than the amount of ingestion at each time point. There were statistically significant (*p* < 0.05) at 5 days after poisoning. The optimal cutoff point was 1.05 ug/mL (AUROC = 0.971, sensitivity = 100%, specificity = 62.69%) of plasma DQ concentration at admission at 5 days. But the prognostic value of both decreased significantly 7 days after poisoning。.

**Table 4:** Prognostic value of plasma DQ concentration on admission and ingestion amount on prognosis.

Indices	Plasma DQ concentration on admission			Ingestion amount		
	AUC	Lower 95%	Upper 95%	AUC	Lower 95%	Upper 95%
1d	0.983	0.957	1.008	0.940	0.880	1.000
2d	0.982	0.956	1.007	0.948	0.899	0.998
5d	0.971*	0.940	1.001	0.885	0.812	0.958
7d	0.960	0.919	1.000	0.869	0.780	0.958
14d	0.835	0.648	1.023	0.582	0.201	0.964

## 4 Discussion

DQ, as a non-selective bipyridinium herbicide, belongs to moderately hazardous (class II) technical grade active ingredients in pesticides according to the WHO recommended classification of pesticides by hazard and guidelines for classification (2019) (17). In the present study, the mortality rate for acute DQ poisoning in hospitalized patients was 36.25% (29/80). The longest survival time of 4.5 days after DQ poisoning, with 23 of the 29 patients dying in 2 days.

In the present study, blood purification was used to promote the excretion of absorbed poisons, but there were no significant differences in different treatment regimens, which is consistent with previous studies (18–21). Only one study indicated that early, repeated hemoperfusion combined with hemodialysis significantly improves blood-gas indices and liver and kidney function in patients with PQ poisoning, also extending their short-term survival. This finding suggests that combining these two purification methods could be more effective than using either method alone, particularly when initiated early and repeated as needed (22). However, it’s important to note that the overall effectiveness of these treatments can vary based on several factors, including the severity of the poisoning, the amount of DQ ingested, and the timing of the treatment initiation. For a comprehensive understanding of their comparative effectiveness, more targeted research specifically comparing hemoperfusion, hemodialysis, and hemoperfusion plus hemodialysis in DQ poisoning would be needed.

The result showed that the plasma DQ concentration on admission is a time-dependent covariate. As a result, we present a time-dependent Cox regression model for the estimate of DQ poisoning prognosis. According to multivariate Cox analysis, plasma DQ concentration at admission and PSS within 24 h after admission were independent prognostic factors for in-hospital death in patients with acute oral DQ poisoning. Plasma DQ concentration at admission and T_COV_PDQ were statistically significant, indirectly suggesting its departure from the proportional hazards assumption. We found that the HR of plasma DQ concentration decreases with varying time (≤1.5 days) due to the negative values of the regression coefficients (coef) of T_COV_PDQ. This result is probably related to the toxicokinetic characteristics of DQ in patients (3). PSS is a simple, less time-consuming, and effective evaluation scale to predict the severity and mortality of poisoning in emergency (23). In the present study, PSS was treated as a continuous variable. For each increase in PSS units, the rate of in hospital fatality hazard increased by 3.47 on the original scale.

The amount of ingestion is often used as an indicator to determine the degree and prognosis of the disease (3, 4, 22). However, the amount of ingestion is often greatly affected by subjective wishes of patients and doctors. To obtain relatively accurate ingestion amounts, this study was performed by two specially trained staff members to assist patients in reproducing the ingestion amount of DQ. The amount of DQ ingestion was 50 (80) mL. Furthermore, ingestion amounts from non-survivors were significantly higher than those of survivors (*p* < 0.001). However, it was not an independent prognostic factor in the multivariate Cox regression analysis. According to time-dependent ROC analysis, plasma DQ AUROC concentration at admission was greater than the amount of ingestion at each time point and was statistically significant (*p* < 0.05) at 5 days after poisoning. These results indicate that the plasma concentration of DQ on admission was higher than the amount of ingestion to predict the survival or death of the patient with DQ poisoning. In this study, the longest survival time was 4.5 days after DQ poisoning and no events occurred beyond 5 days. This may be related to the fact that most of the non-survivors were fulminant poisoning.

Using a cut-off value (1.05 ug / mL, AUROC = 0.971, sensitivity = 100%, specificity = 62.69%) of plasma DQ concentration at admission 5 days after poisoning, the cumulative survival rate of the high concentration group (≥1.05 ug / mL) was only 17.1%. Therefore, in patients with a plasma DQ concentration greater than 1.05 ug/mL taken within 36 h after ingestion, the prognosis is poor. And neither HP nor HP combined with CVVH could improve target organ damage. However, for patients with plasma DQ concentrations below 1.05 ug/mL taken within 36 h after ingestion, HP and (or) CVVH should be actively administered early to increase toxicant excretion, reduce target organ damage, and improve patient prognosis.

## 5 Limitations

There are several limitations and strengths in our study. First, selection bias or unmeasurable confounding factors may exist in the observational study. For example, we could not measure other factors potentially associated with mortality, such as other medications use, imaging reports, or genetic biomarkers that might affect a provider’s treatment decision-making. Second, the present study is a single-center retrospective study. The sample size is rather small for patients with acute oral DQ poisoning for statistical analysis. A multi-center clinical study is required. Third, most of the non-survivors were fulminant poisoning and died within 2 days after poisoning, potentially leading to bias. Finally, due to ethical considerations, all patients included in this study were treated with blood purification. Although this study does not prove that blood purification can affect the prognosis of patients, its efficacy still needs to be further explored.

## 6 Conclusion

By using statistical tests and graphical diagnostics based on scaled Schoenfeld residuals, the plasma DQ concentration at admission and poisoning severity score within 24 h after admission are independent prognostic factors for the in-hospital case fatality rate in patients with acute oral DQ poisoning. The prognostic value of plasma DQ concentration decreased with time. These findings, in conjunction with the results of other studies showing associations of the plasma DQ concentration with the prognosis, may help physicians make better decisions for acute DQ poison treatment.

## Data availability statement

The raw data supporting the conclusions of this article will be made available by the authors, without undue reservation.

## Ethics statement

The studies involving humans were approved by The Hebei Medical University Institutional Review Board. The studies were conducted in accordance with the local legislation and institutional requirements. The participants provided their written informed consent to participate in this study.

## Author contributions

NM: Formal analysis, Methodology, Writing – original draft, Writing – review & editing. YS: Writing – review & editing. YT: Funding acquisition, Investigation, Project administration, Writing – original draft, Writing – review & editing. YD: Writing – review & editing. BL: Writing – review & editing. DY: Writing – review & editing. HG: Writing – review & editing. YM: Writing – review & editing. YJ: Writing – review & editing. TZ: Writing – review & editing.
